# Removal of manganese from wastewater using Moringa stenopetala plant parts as an adsorbent material

**DOI:** 10.1016/j.heliyon.2023.e22517

**Published:** 2023-11-21

**Authors:** Ashenafi Zeleke Melaku

**Affiliations:** Department of Chemistry, Faculty of Natural and Computational Sciences, Woldia University, PO. Box 400,Woldia, Ethiopia

**Keywords:** Bioadsorbnt, Enviromental friendly, Industrial waste, Isotherm, Kinetic

## Abstract

Removal of heavy metal ions from industrial effluents using environmental friendly bioadsorbents is currently promising approach. However, removal of manganese metal ion via *Moringa stenopetala* (*M.stenopetala)* plant material is not studied yet. Thus, parts of the plant has been studied as bio adsorbents for removing toxic manganese ion from aqueous solutions in batch adsorption model. The maximum percent removal of manganese ion obtained from laboratory synthetic wastewater at equilibrium are 96.05 %, 98.90 % and 97.93 % by *M. stenopetala* plant leaf, bark and seed, respectively. However, the use of *M. stenopetala* plant leaf procedures an intensive color with unpleasant odor, which is inauspicious. Therefore, *M. stenopental plant leaf* was no longer examined for isotherm and kinetics studies. The fitness of adsorption data were confirmed based on the value of correlation coefficient (R^2^). Thus, adsorption by bark best fits of Temkin model with R^2^ value of 0.9707, while adsorption by seed follows the Langmuir model with R^2^ value of 0.9733. Adsorption kinetics result indicates that pseudo second-order model well fitted with R^2^ value of 0.9912 and 0.9947 for bark and seed adsorbents, respectively. Additionally, the applicability of laboratory-developed method was also evaluated on a multicomponent real sample taken from KK textile industry from Addis Abeba, Ethiopia. After characterization, the percentage removal of manganese ion were 79.53 % and 88.93 % for bark and seed, respectively. This achievement is promising and in a good agreement with the results of single component laboratory synthetic wastes.

## Graphical abstract

**Figure:**
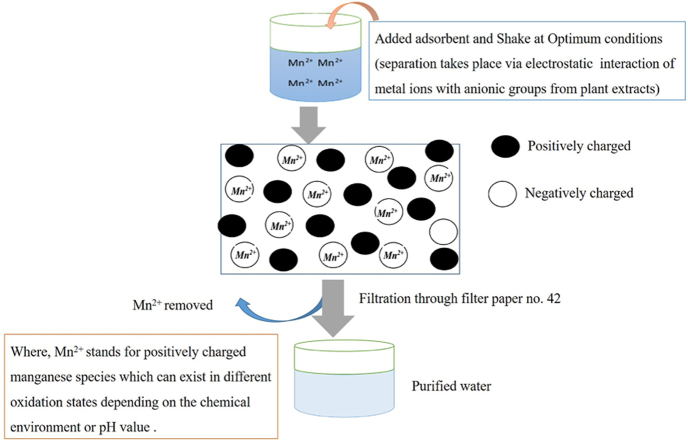

## 1 Introduction

Environmental deterioration has been mostly attributed from industrial wastes. Industrial waste dispose into enviromental segments is a significant issue that affects the biota in the receiving environment. Heavy metal concentrations in industrial waste effluents from the metal-finishing and metallurgical sectors are frequently high and pose major environmental pollution issues. One of the biggest issues is heavy metal contamination into waterbody and soil. In addition, toxic metal compounds came into contact with earth's surface can also leach into underground water after rain and snowfall. Consequently, several harmful metals may be present in the earth's water as well [1,2]. The building-up of these metals in food structure is one of the most significant issues. Its concentrations in foodstuff can be higher than those in water and air due to accomulation. Food poisoning from the tainted food can affect all living things. Although some heavy metals are essential for plant growth, they will harmful above threshold limits and hence, it is crucial to look for inventive ways to prevent environmental pollution. Therefore, strategies and methods introduced to reduce the pollution of these metal causes are appealing [[3], [4], [5]]

Heavy metals and harmful compounds can be removed from industrial effluents using a variety of techniques, including chemical precipitation, ion exchange, coagulation/flocculation, reverse osmosis, and others [[6], [7], [8]]. The majority of the aforementioned procedures do, however, have certain disadvantages, like low levels of metal removal, high energy consumption and reagent usage, produces toxic sludge, etc. [9]. However, the use of enviromental friendly bioadsorbents are an alternative approach that reduces most shortcomings of other techeniques. Adsorption is by far the most adaptable and efficient technique for removing pollutants such as heavy metals, dyes especially when used in conjunction with the proper regeneration stages. This resolves the sludge disposal issue and improves the system's economic viability. In this approach, use of bio-adsorbents instead of chemicals to remove heavy metals from industrial effluents is a great idea reported recently. *M. stenopetala* plant parts including bark and seed were reported that contains different functinal groups/phytochemicals like OH-, amines, carbonyls, sulphates, arromatics, and the like which is responsible for adsorpative removal of metal ions from industrial influents [[10], [11], [12], [13]].

Nowadays, there is a wide expansions of industries established for the manufacture of processed goods along with excessive wastes throughout Ethiopia. However industrial wastes are not successfully treated before disposed into the nearby environmental segments. The economy of the country is not strong enough to use more advanced waste treatment technology. As a result, there is an urgent need to develop low-cost, effective and biofriendly adsorbents that effectively remove wastes. In line with this, there are reports that natural adsorbents constitute an excellent alternative for chemical remediation of heavy metals from industrial effluents [14]. Kebede and Colleague in (2018) reported a successful adsorption removal of copper, cadmuim and lead from synthetic wastewater using moringa stenopetal plant parts [10,11]. However no report about manganese ion removal by the plant parts yet. Therefore the aim of this study was curious to investigate the adsorption efficiency of *M.stenopetala* seed and bark as adsorbent material for manganese ion adsorption removal in batch adsorption model.

The main objective of this study was to investigate manganese ion removal efficiency of *M*. *stenopentala* plant materials from wastewater. The results made by this study confirms excellent, enviromental friendly and inexpensive adsorbents for removal of manganese metal ion from industrial effluents.

## 2 Materials and methods

*M.stenopetala* plant samples (leaf, bark and seed) were collected from koso share village, near Arbaminch in Southern Ethiopia, which is 500 km from Addis Ababa. *M.stenopetala* tree leaf, bark and seed were washed by distilled water to remove any contaminates. Then, seed was dried for 24 h at 110 °C and bark was dried at 105 °C in an oven overnight [7]. The dried samples were ground by mortar and pestle and sieved by 0.5 mm siever to get particle size less than or equal to 0.5 mm^,^ [15].

### 2.1 Preparation of adsorbate solutions

Analytical-grade reagents and deionized water were used in this study. 1000 mg/L of a synthetic stock solution of manganese was prepared using MnSO_4_.H_2_O in double distilled water [4,16]. The working solutions were obtained by diluting the stock solution with double distilled water. The pH adjustment was carried out using diluted HCl and NaOH.

### 2.2 Characterization of adsorbent materials

The *m.stenopetala* plant parts characterization taken from Arbaminch, Ethiopia confirmed the availablity of phytochemicals with a wide ranges of organic functional groups as reported Kebede and his coworkers in 2018 [10,11]. Having this informations in mind, the functional groups binding ablity of manganese ion from both single and multicomponent system were studied.

### 2.3 Batch adsorption procedure

The batch experiment was performed by adding desired amount of metal solution in 50 mL volumetric flask at desired adsorbent dose, pH, agitation speed and temperature. The solution was transforrmed in to 250 mL conical flask and shaken by mechanical shaker (Digital RJH5005 12-Stand Orbital Shaker) at desired rpm for definite periods. Adsorbent dose, metal ion concentration, contact time, agitation speed, pH, and temperature were optimized by continuous variation approach (studying one parameter keeping the others constant) [17]. The difference in the metal ions concentration before and after adsorption represents the metal ion adsorbed and it was measured using flame atomic absorption spectroscopy (ELICO SL 194, India). The FAAS was adjusted to measure each sample five times and the results taken as an average.

### 2.4 Collection and characterization of real wastewater

Wastewater samples were collected in triplicates from local industries found in Addis Abeba, KK textile industry. Three waste water samples were colleted from the discharge point of the effluent using preclear and acidified plastic bottles with a 30 min interval and it was mixed together. The sample was then, kept in ice bag and transported to laboratory for analysis.

### 2.5 Data analysis

The percent of metal removed, R, was calculated according to Mahmoud, A. E. *and coworkers* (2020) [18] given as equation (1) follow:

1 $$\%R=\frac{C_{i}-C_{e}}{C_{i}}x\;100$$

Where, C_i_ and C_e_ being the initial and metal concentration in mg/L at equilibrium, respectively.

For isotherm and kinetic study the data were taken at the equilibrium conditions using the equations dislayed under Table 1. The amount of metal ions adsorbed per unit mass of the adsorbent at equilibrium, *q*_*e*_ (mg/g), was calculated using equation (2) as indicated in Chen X. and his colleagues *in* 2021 [19,20].

2 $$q_{e}=\frac{\left(C_{0}-C_{e}\right)V}{m}$$

where, $q_{e}$ is the equllibrium adsorption capacity, volume of aquous solution taken and m is mass of adsorbent used.

**Table 1:** Adsorption isotherm and kinetics models were computed using the following kinetic/adsorption isotherm model equations [21,22].

kinetic/adsorption isotherm model	Linear equation
Freundlich	logqe=logk+1nlogCe=kf+1nlogCe
Langmuir	$\frac{C_{e}}{q_{e}}=\frac{1}{Q_{o}b}+\frac{C_{e}}{Q_{o}}$ and $R_{L}=1/(1+bC_{e}$
**Temkin Isotherm**	$q_{e}=BlnA+BlnC_{e}$
**Pseudo First Order**	ln($q_{e}-q)=\ln\;q_{e}-k_{1ad}t$
**Pseudo Second Order**	$\frac{t}{q_{t}}=\frac{t}{k_{2ad}q_{e}^{2}}+\frac{1}{q_{e}}t=\frac{1}{h}+\frac{1}{q_{e}}t$
**Elovich**	$q_{t}=\frac{1}{\beta }$ ln(αβ) + $\frac{1}{\beta }\ln\left(t\right)$
**Interparticle diffussion**	$q_{t}=\frac{C_{t}V}{mx100}xK_{id}t$^a^

## 3 Result and discussion

*M. stenopetala* plant parts have different kinds of phytochemicals responsible for the adsorption metal ions easily from metal ion containing waste solution [6]. Adsorption capablity of the selected plant parts were evaluated using batch adsorption.each of the selected adsorption parameters were optimized consquetively as presented below.

### 3.1 Effect of initial concentration of metal ion

The % adsorption studied by varying the initial adsorbate concentration from 5 50 mg/L.

The optimum adsorbate concentration for the adsorption process by leaf, bark and seed were 40, 10 and 30 ppm with maximum percent adsorption of 87.65 %, 92.20 % and 93.10 %, respectively.

**Fig. 1:**
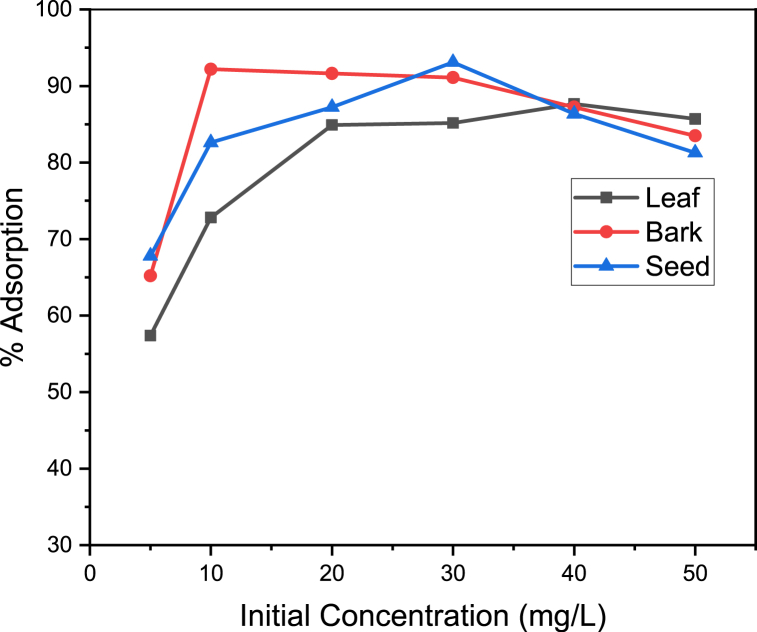
Adsorption (%) versus initial concentration of manganese.

The variation in the amount of manganese ion bound to the adsorbents with increasing initial metal ion concentration is illustrated in Fig. 1 above.

The increase in adsorbate's concentration caused a rise in the percentage of agsorption intially. Since adsorption process is dependent on the surface area of the adsorbent, the initial quicker removal of the metal ions may have been caused by the availability of the exposed free surface area of the adsorbents. With time, more adsorption sites were being covered as the metal ion concentration raised. Additionally, higher starting concentrations increased the metal ion's affinity for the active sites [23,24]. A logical explanation for the decline in the % adsorption is due to lesser surface sites available on the adsorbent surface. In this regard Bhuptawat and his co-authers reported that the removal of Cd (II) from aqueous solution using powdered shelled *Moringa oleifera seed*, is similar with this findings [25].

### 3.2 Effect of adsorbent dose

The effect of adsorbent dose and the percentage adsorption of the metal ions studied by varying the adsorbent dose from 0.5 3.5 g.

**Fig. 2:**
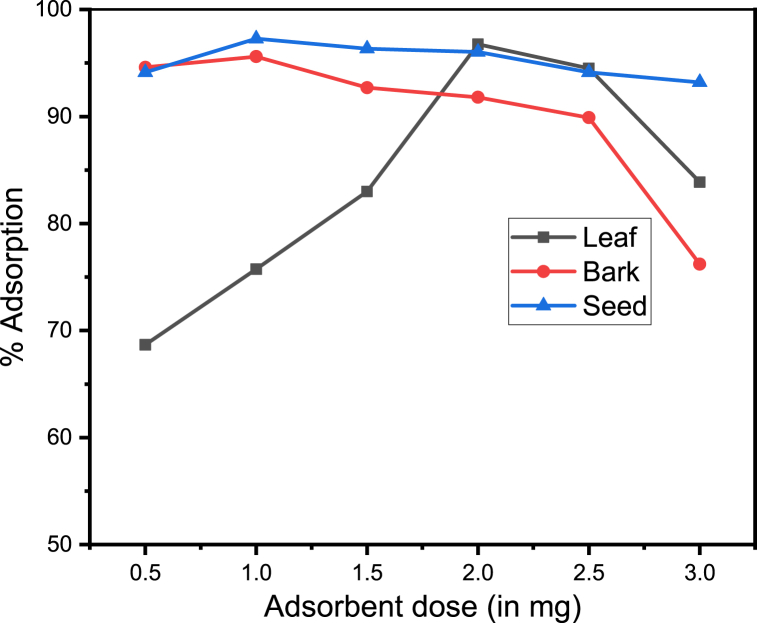
Adsorption (%) of manganese versus Adsorbent dose The optimum adsorbent dose of leaf, bark and seed were 2, 1 and 1 g with maximum percent adsorption of 96.75 %, 95.60 % and 97.27 %, respectively.

The quantity of the adsorbent is one of the factors that significantly affect adsorption capacity. It is clear from Fig. 2 that the adsorption extent increases with adsorbents' weight. This confirms free active sites of adsorbent have a greater availability of exchangeable sites for manganese ion. A similar result was also reported by Oboh, O. and his collegues about biosorption of metal ions [26]. Due to the increase in adsorbent dose, there is an increase in the percentage adsorption but at a marginal rate. It was observed that the percentage adsorption had increased up to the optimum dose. Even though, the total metal uptake increase with the dose of adsorbent, initially, it decreases after the attainment of the optimum dose. This might be agrregation of excess adsorbent dose overlap, and overcrowding reduce the numbers of active sites on the surface [27].

### 3.3 Effect of pH

The optimum pH value was obtained though varying its value while keeping constant other parameters. Within the pH range of 1–7.5, the impact of the initial pH value on the removal of manganese ions by the adsorbent was examined.

**Fig. 3:**
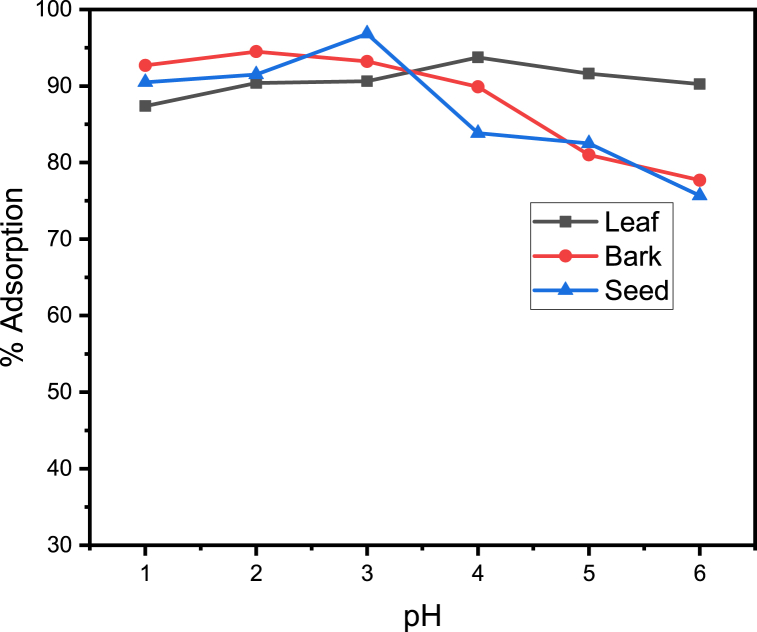
Adsorption (%) of manganese versus pH The optimum pH at which maximum adsorption of manganese ion by leaf, bark and seed with maximum percent adsorption of 93.75 %, 94.50 % and 96.83 %, respectively.

The protonation and deprotonation kinetics of acidic and basic groups would vary at various pH values. The metal ions would have a different surface structure than the adsorbent. Fig. 3 displays the experimental findings concerning the influence of pH on the non-competitive adsorption of manganese ion. For each of the adsorbents, it can be observed that adsorption increased as pH increased. However, as the pH of the solution rises even higher, more OH- is produced, which interactes with the manganese ion forming precipitates. However, positively chargesd manganese ion electrostatically reppelled by the large hydrogen ion at the interface at low pH and thus prevented from approaching to the adsorbent surface. Similar phenomenon of cobalt adsorption using *Amaranthus hydridus* L. stalk wastes reported by Egila, J. N and his collegues [28].

#### 3.3.1 Effect of contact time

The percentage adsorption with different adsorbate concentrations studied by varying the contact time from 30 240 min.

**Fig. 4:**
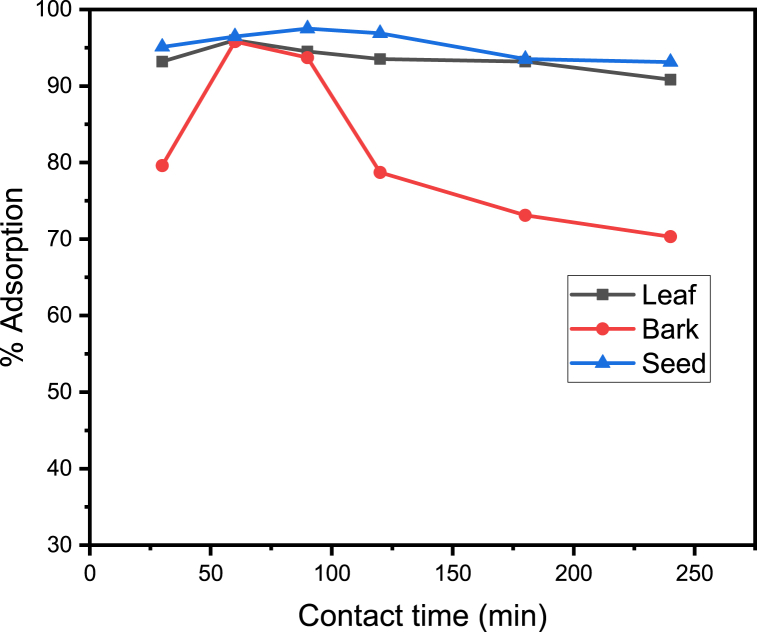
Adsorption (%) of manganese metal ion versus Contact time.

The optimum contact time for the adsorption of metal ion by the adsorbent leaf, bark and seed are 60, 60 and 90 min with the maximum percent adsorption of 96.00 %, 95.80 % and 97.50 %, respectively. As shown in Fig. 4 above, increase in adsorption extent with contact time can be attributed to the fact that metals need time to reach the active sites of the adsorbent surface. Initial adsorption occurs immediately as soon as the metal and the adsorbent came into contact. But after that when some of the easily available active sites occupied, metal needs time to find out more active sites for binding. This finding confirms that the binding of Manganese ion was found to be less within the initial contact time. But later on, the percentage adsorption of the metal ion reaches maximum and attained equilibrium by the end of the suitable contact period. This implied that the binding sites were exhausted and further shaking resulted in desorption. These results are in a good agreement with the report of Egila, J. N and his collegues [28].

### 3.4 Effect of agitation speed

Effect of agitation speed on adsorption of metal ions was studied by varying the agitation speed form 100^_^350 rpm.

**Fig. 5:**
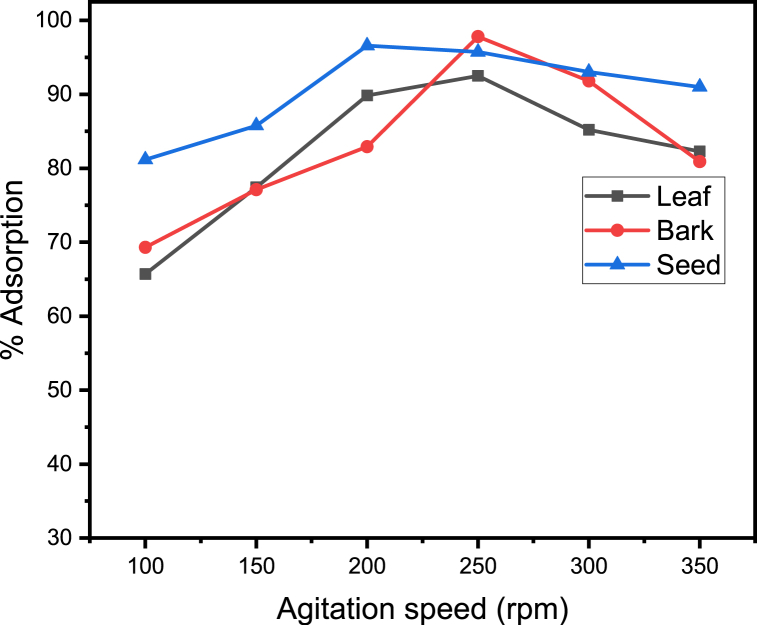
Adsorption (%) of manganese ion versus Agitation speed.

The optimum agitation speed of adsorption by leaf, bark and seed of the adsorbent are 250, 250 and 200 rpm with maximum percent adsorption of *92.48 %, 97.80 %, and 96.57 %, respectively.* Fig. 5 shows that the adsorption is strongly impacted by the shaking speed. Adsorption increases as shaking speed rises. If the shaking is done slowly, the adsorbent builds up together rather than spreading, and different active sites are buried behind the layers above. Therefore, only the uppermost layers are removed, and the lower layers, which are not in contact with the metal, are not involved in the process. This suggests that a steady shaking rate should be adequate to ensure that all of the surface active sites are open to metal uptake. But, when the agitation speed gets fast beyond the steady shaking rate, manganese ion adsorption falls down, due to collision of metal ions with each other before reached to the active sites resulting desorption. The finding of this study has a good agreement with the reports of Kebede T.G and his collegues [10,11].

## 4 Effect of temperature

The effect of the temperature on the adsorption of manganese ion onto the prepared adsorbent studied within the range 23–60 °C.

The optimum temperature for adsorption of manganese by leaf, bark and seed are 30, 30 and 23 °C with maximum percent adsorption of 96.05 %, 98.90 % *and* 97.93 %, *respectively.*

**Fig. 6:**
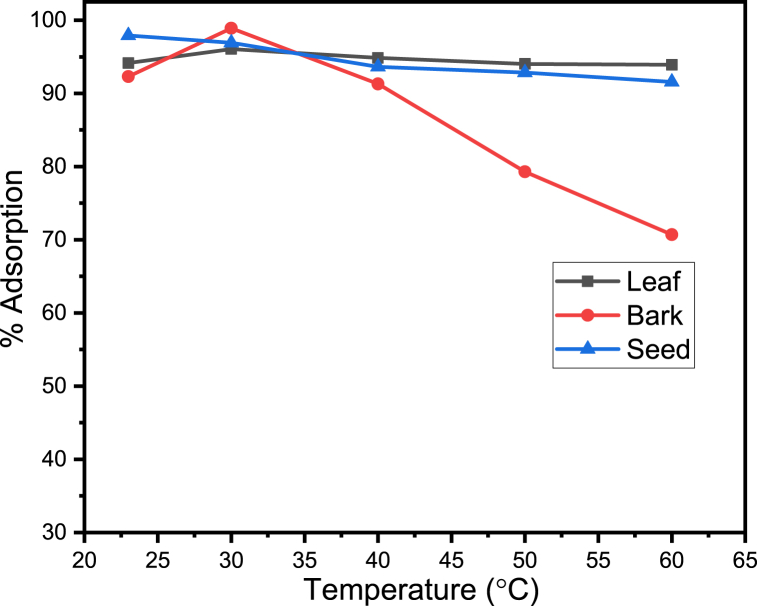
Adsorption (%) of manganese metal ion versus Temperature.

The results reported in Fig. 6 clearly illustrate that when the temperature rise, the amount of metal ion that bark could adsorb increased marginally. This may be as a result of very slow adsorption steps being accelerated or the development of additional active sites on the surface of the adsorbent. There are two key impacts of temperature on the adsorption process. One is that, increasing temperature speeds up the rate of adsorbate diffusion across the external boundary layer and inside the pores of adsorbent. On the otherhand, it affects the adsorbate's equilibrium capacity depending on whether the process is exothermic or endothermic.This finding is inline with other ' findings, including uses of Ashoka Leaf Powder for nikel, cadmium and iron adsorpitive from aqueous solution reported by Shelke R. S. and his colleagues [29].

A subsequent reduction in adsorption capacity observed when the temperature further raised to allow the metal ion to be absorbed by the seed and leaf. This might be losening of attraction force between manganese ion and adsorpent active sites and the manganese ion are more likely to escape from the biomass surface and enter the solution phase at high temprature. The result of increasing temperature is a decrease in adsorption [31].

### 4.1 Comparison of different parts of the adsorbent

The adsorption of manganese ion from aqueous solution were presented below.

From the batch adsorption study: result indicated in Table 2 confirm that the three parts of the *M.stenopetala* tree are suitable for the removal of manganese ion possibly present from industrial effluents. But in comparison with each other, *M.stenopetala* bark and seed fits well. Beside this, even if *M.stenopetala* leaf is well fitted to remove manganese ion present in industrial effluents, it is intensively colored and has bad odor. Due to color and bad odor formation, the leaf does not use as waste treatment plant part unless some modification is made on it. Therefore, *M.stenopetala* bark and seed were selected as well suited adsorbents of manganese ions in industrial effluents. Though all the parts are effective adsorption phenomenon, the order of adsorption at optimum conditions are (%) are bark(98.90 %) > seed (97.93 %) > leaf (96.05 %).

**Table 2:** Comparison of leaf, bark and seed of *M.stenopetala* tree on their percent adsorption.

Adsorbents	% Adsorption
**Leaf**	**96.05**
**Bark**	**98.90**
**Seed**	**97.93**

## 5 Adsorption isotherms

**Fig. 7:**
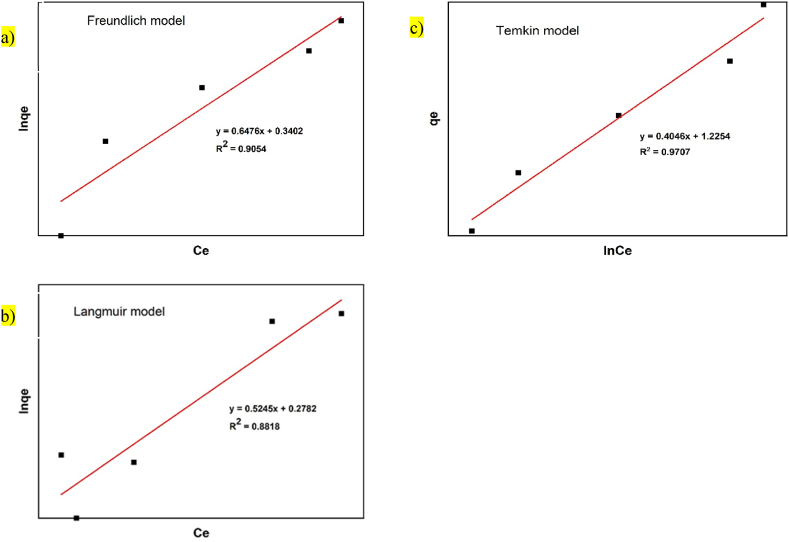
Plot of a) -8Freundlich b) Langmuir and c) Temkin isotherm models of Ce/qe Vs Ce, lnqeVs lnt and qe Vs lnt by bark, respectively. *′*

**Fig. 8:**
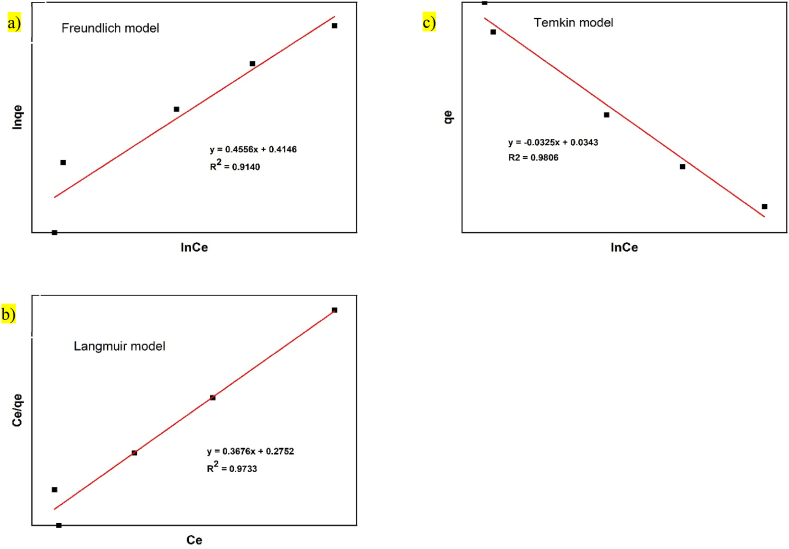
Plot of a) Freundlich b) Langmuir and c) Temkin isotherm models of Ce/qe Vs Ce, lnqeVs lnt and qe Vs lnt by seed, respectively.

To find the most appropriate model, the data were fitted to different isotherm models and computed using equations presnted in Table 1. The obtained parameters values are shown in Table 3 and Fig. 7, Fig. 8. as well. The values of R^2^ are regarded as a measure of the goodness-of-fit of experimental data. Also values of R_L_ found to be between 0 and 1 indicating that both adsorbents are favorable for adsorption of Manganese ion from aqueous solution. Additionally, the adsorption intensity (n) is greater than unity implies that the forces within the surface layer are attractive confirming manganese ion is favorably adsorbed by *M.stenopetala* plant (bark and seed) [32,33]. Particularly, it is evident that the equilibrium data best confirm to the Freundlich model. This observation is consistent with the heterogeneous nature of the adsorbent surface, which consists of different active sites.

**Table 3:** Parameters of Langmuir Temkin and Freundlich adsorption isotherm for Manganese ion *on M. stenopetala* adsorbent at different initial concentration.

Isotherm model	Parameters	Adsorbent	
		Bark	Seed
Freundlich	K_F_ n R^2^	1.4052 1.5442 0.9054	1.5138 2.1954 0.9140
Langmuir	Q_0_ b R_L_ R^2^	1.9066 1.8853 0.8414 0.8818	2.7203 1.3358 0.6044 0.9733
Temkin	A B R^2^	20.6697 0.4046 0.9707	10.9489 0.6477 0.9806

As shown in Table 3 below, the *R*^2^ value of the isotherms showed both isotherms fit well. In comparison with each other, the Langmuir model was able to adequately describe the adsorption of Manganese ion by seed with R^2^ value of 0.9733. While, Temkin model successfully describes the adsorption of Manganese ion by bark with R^2^ value of 0.9707, which reflects the occupation of the more energetic adsorption sites at first. It is highly probable that their adsorption sites are energetically non-equivalent. Similar results were also reported in previuos studies [17]. The adsorption isotherm model fitness is different for bark and seed of *M.stenopetala*. This is obviously may be because of the surface morphology and functional groups available on each part of *M.stenopetala* plant.Where, $K_{F}$ = Freundlich isotherm constant ((mg/g (L/mg) ^1/n^), n = Freundlich Adsorption tendency,

$Q_{o}$ = Langmuir isotherm constant (mg/g), b = Langmuir isotherm constant (L/mg),
$R_{L}$ = Dimensionless constant separation factor, A = Initial adsorption rate of Temkin model,
B = Related to the occupied surface of Temkin model

## 6 Adsorption kinetics

To ascertain the adsorption kinetics of heavy metal ions, the kinetics parameters for the adsorption process were evaluated for various contact times by measuring the metal ion % adsorption. Following that, the data were regressed by different kinetics models, such as the Elovich kinetics equation, the Intraparticle diffusion kinetics equation, pseudo-first order kinetics equation and a pseudo-second order kinetics equation as presented in Table 1 above, respectively. The experimental results indicates that the pseudo first and pseudo second order kinetics equations are accord quite well. Relatively, the entire experimental data is best suited by a pseudo-second order kinetic equation. The value of different adsorption kinetic parameters were computed and the results are shown belowin Table 4.

**Table 4:** Parameters of computed kinetics models for different adsorbent types.

kinetics	Parameters	Adsorbent	
		bark	seed
Pseudo 1st order	K_1ad_ q_cal._ R^2^	0.0188 6.2546 0.8480	0.0263 1.4581 0.9184
Pseudo 2nd order	K_2ad_ q_cal._ R^2^	0.716 0.947 0.9912	0.205 0.903 0.9947
Elovich	α β R^2^	3.18 × 10^−7^ 85.4701 0.0716	6.90 × 10^−8^ 10.0200 0.5052
Intraparticle diffusion	a k_id_ R^2^	0.0267 0.4999 0.0764	0.0707 1.9599 0.5097

$K_{1ad}$ = Equilibrium rate constant of pseudo-first sorption (1/min), $q_{cal}$_.=_Amount of metal sorbed (theoretical) mg/g, $K_{2ad}$ =Equilibrium rate constant of pseudo-second sorption (g/mg min), a = Gradient of linear plots of intraparticle diffusion model, β = occupied surface, in g/mg, α = the initial adsorption rate, in mg/g·min, $K_{id}$ =Intraparticle diffusion rate constant.

The value of correlation coefficient, $R^{2}$ can be used to determine the degree of goodness in linear plot of kinetic models. From the determination of R^2^ value indicated in Table 4 below, adsorption of Manganese ion on the *M. stenopetala* bark and seed is regarded as pseudo-second order kinetics. Similar results were also found in Refs. [20,31]. The adsorption kinetics model fitness is different for bark and seed of M.stenopetala. This is obviously may be because of the surface morphology and functional groups available on each part of *M.stenopetala*.

### 6.1 Proposed adsorption mechanism

**Fig. 9:**
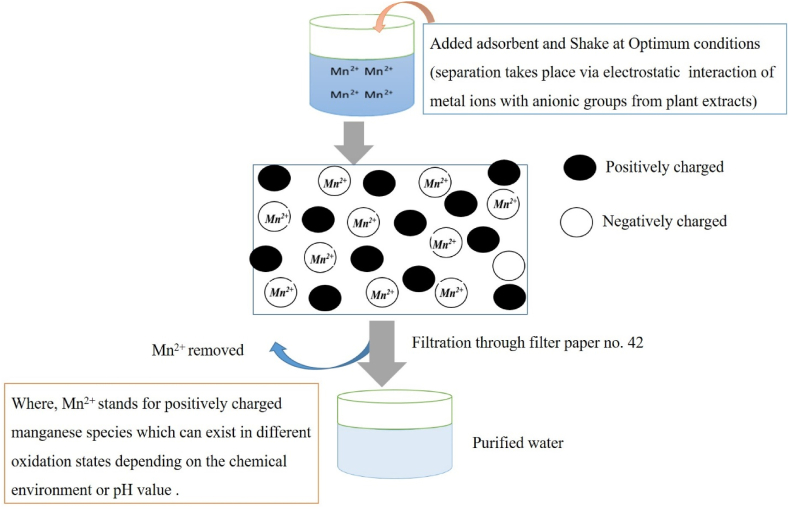
Shows the proposed mechanism for manganese metal ion adsorption on an adsorbent's surface via physical and chemical interaction and its separation by filtration as supported by Rudi, N.N and his coworkers [20,30].

The adsorbent materials have various types of phytochemicals with multifunctional groups as well as surface morphologies, according to previous study reports on the adsorption phenomena [10,11,13,20]. In order to maintain its position, the positively charged manganese ion intercalated itself inside the available adsorbent pore and also held with negatively charged groups by electrostatic force [21] Fig. 9.

### 6.2 Application of the developed method to real sample

The utilization of the *M.stenopetala* tree (bark and seed) as an adsorbent material was assessed by its application in treatment of industrial waste water sample. Textile industry waste water samples containing the selected metal ions were collected from local industries situated in Addis Abeba, KK textile industry. The physicochemical characteristics of industrial wastewater that could affetc adsorption process was studied as shown in Table 5 below.

**Table 5:** Physicochemical characteristics of wastewater samples from KK textile industry.

Prameters	Analysis method	Analysis value
pH Total dissolved solid(TDS) Conductivity Total solids (TS) Total suspended solid (TSS) Temperature Salinity (Conductivity ratio) Alkanity Chemical oxygen demand (COD) Dissolved oxygen (DO) Sulphate Manganese, Mn(II)	pH-meter TDS- meter Conductivity meter Gravimetric Gravimetric Thermometer Conductivity meter Titration Titration DO- meter Gravimetric AAS	6 470 mg/L 740 μS/cm 1472 mg/L 732 mg/L 40 °C 0.2 0.02 mg/L 280 mg/L 4.8 mg/L 996.48 mg/L 5.96

The evaluation of laboratory developed method revealed that the adsorption capacity is not significanty different from the results obtained based on single synthetic wastewater solution experiments. Thus, this tudy demonstrates that *M.stenopetala* can be successfully used for the removal of selected metal ion from multicomponenet system real waste.

### 6.3 Real application of selected adsorbent material

Application of *M.stenopeta* bark and seed as adsorbent materials for the removal of manganese ions from industrial effluents were evaluated as shown in Table 6 below. This experiment was conducted to study manganee ion removal efficiency by selected adsorbents from a complex compositions of industrial enffluents. This was done under optimized parameters using laboratory synthetic waste water.

**Table 6:** Application of *M.stenopeta* bark and seed as adsorbent materials for the removal of manganese ions from real sample or industrial effluents on the optimum conditions.

Adsor-bent	Metal	C_i_ (mg/L)	C_e_ ± SD (mg/L)	% R
Bark	Mn	5.96	1.22 ± 0.02	**79.53**
Seed	**Mn**	**5.96**	**0.66 ± 0.02**	**88.93**

From this result, it is possible to suggest adsorbent effectiveness of manganese metal ion holding the optimum conditions obtained using laboratory prepared synthetic wastewater. *M. stenopetal* plant bark remove about 79.53 % of manganese ion from real industrial wastewater taken from KK textile industry, in Addis Abeba, Ethiopia. In the other hand the seed adsorption is 88.93 % of manganese from same real wastewater taken from KK textile industry. According to the obtained results on adsorption removal of mananese ion from KK textile industry, the seed is more efficient in adsorption of manganese ion than the bark (seed- 88.93 % > bark −79.53 %). It shows a slight decrease of adsorption efficiency comparing to single manganese ion containing synthetic wastewater due to computation of other ions from real wastewater to occupy adsorption sites. This result indicates that *M.stenopetala* plant parts are a suitable cheap and effective adsorbent for the removal of manganse metal ions from industrial effluents.

Adsorption efficiency of *M. stenopetala* bark and seed were compared with literature values shown in Table 7. Various kind of adsorbents were applied for adsorption of heavy metals from aqueous solution. Comparing to some of the reports presented below confirmed the promising *M. stenopetala* bark and seed adsorbents for remediation of environment from manganese pollution. The antagonistic effect of heavy metal removal under ideal circumstances was confirmed by the results in Table 7.

**Table 7:** Comparison of current study with literature values reported.

Metal ion	Adsorbent	% Adsorption	Reference
Cd	*M.stenopetala* bark and seed	94.8 and 94.17, resprctively	[10,11]
Pb		95.5 and 94.67, resprctively	
Cu		94.23 and 92.80, resprctively	
Pb, Cd, and Zn	*Gossypium barbadense*	98, 92.1, and 78.9, respectively	[34]
	*Phoenix dactylifera*	94.6, 76 and 68.6, respectively	
Mn	palm fruit bunch	76	[35]
Mn	maize cob	79	
Mn	Activated Rice husk	79.6	[36]
Mn	Papiliotrema huenov	60.3/hr	[37]
Mn	*M.stenopetala* bark	79.53	This work
Mn	*M.stenopetala* seed	88.93	This work

## 7 Conclusion

This study evaluated the removal of manganese ion from synthetic wastewater using a low-cost and effective ***M.stenopetal*** plant bark and seed adsorbent materials in lab scale. The maximum adsorption efficiency of bark and seed using single component synthesic wastewater are 98.90 % and 97.93 % while in multicomponent real wastewater are 79.53 % and 88.93 respectively. Based on the obtained results, the researcher concluded that the bark and seed of *M.stenopetala* plant parts are effective adsorbents for remoal of manganese ion from industrial enffluents. This study found that adsorption parameters such as pH, contact time, adsorbent dosage, temperature, and stirring speed affect Manganese ion adsorption. The equilibrium data were applied to the Langmuir, Temkin and Freundlich isotherm models and pseudo first order, pseudo second order kinetics, Elovich and Intraparticle diffusion kinetics model. It was found that the adsorption of manganese by bark well fitted to the Temkin isotherm model while Langmuir isotherm successfully describes the adsorption of Manganese ion by seed very well. Among the kinetics models, the pseudo second order kinetic model best fits for the kinetics study that describes the chemosorption mechanisms of adsorption. The developed method is promising in treatment of industrial wastes. In conclusion, this study confirms a potential of environmental friendly *M. stenopetala* plant bark and seed as an adsorbent in removing manganese ion from wastewater. The results obtained are therefore very encouraging for developing industrial application of bio-adsorption technique.

## Funding

No funding was received for conducting this study.

## Data availability statement

The data used to support the findings of this study are included in this report.

## CRediT authorship contribution statement

**Ashenafi Zeleke Melaku:** Writing – review & editing, Writing – original draft, Validation, Project administration, Methodology, Investigation, Funding acquisition, Formal analysis, Data curation, Conceptualization.

## Declaration of competing interest

There is no conflict of interest in this study.
